# Protective Effects of Pituitary Adenylate Cyclase-Activating Polypeptide (PACAP) Against Oxidative Stress in Zebrafish Hair Cells

**DOI:** 10.1007/s12640-016-9659-8

**Published:** 2016-08-24

**Authors:** Natalia Kasica, Piotr Podlasz, Maria Sundvik, Andrea Tamas, Dora Reglodi, Jerzy Kaleczyc

**Affiliations:** 1 Department of Animal Anatomy, Faculty of Veterinary Medicine, University of Warmia and Mazury, Oczapowskiego 13, box 105J, 10-719 Olsztyn, Poland; 2 Department of Pathophysiology, Forensic Veterinary and Administration, Faculty of Veterinary Medicine, University of Warmia and Mazury, Oczapowskiego 13, 10-719 Olsztyn, Poland; 3 Department of Anatomy, Neuroscience Center, University of Helsinki, Haartmaninkatu 8 (Biomedicum Helsinki), 00290 Helsinki, Finland; 4 Department of Anatomy, University of Pecs, Szigeti 12, 7624 Pecs, Hungary

**Keywords:** PACAP, Apoptosis, Antioxidant, Hydrogen peroxide, Neuromast, Behavior

## Abstract

Pituitary adenylate cyclase-activating polypeptide (PACAP) is a pleiotropic neuropeptide, with known antiapoptotic functions. Our previous in vitro study has demonstrated the ameliorative role of PACAP-38 in chicken hair cells under oxidative stress conditions, but its effects on living hair cells is now yet known. Therefore, the aim of the present study was to investigate in vivo the protective role of PACAP-38 in hair cells found in zebrafish (*Danio rerio*) sense organs—neuromasts. To induce oxidative stress the 5-day postfertilization (dpf) zebrafish larvae were exposed to 1.5 mM H_2_O_2_ for 15 min or 1 h. This resulted in an increase in caspase-3 and p-38 MAPK level in the hair cells as well as in an impairment of the larvae basic behavior. To investigate the ameliorative role of PACAP-38, the larvae were incubated with a mixture of 1.5 mM H_2_O_2_ and 100 nM PACAP-38 following 1 h preincubation with 100 nM PACAP-38 only. PACAP-38 abilities to prevent hair cells from apoptosis were investigated. Whole-mount immunohistochemistry and confocal microscopy analyses revealed that PACAP-38 treatment decreased the cleaved caspase-3 level in the hair cells, but had no influence on p-38 MAPK. The analyses of basic locomotor activity supported the protective role of PACAP-38 by demonstrating the improvement of the fish behavior after PACAP-38 treatment. In summary, our in vivo findings demonstrate that PACAP-38 protects zebrafish hair cells from oxidative stress by attenuating oxidative stress-induced apoptosis.

## Introduction

Oxidative stress is a pathological state when reactive oxygen species (ROS), products of normal cellular metabolism, are overproduced and antioxidant defenses are not sufficiently efficient (Valko et al. 2007). Oxidative stress through free radical action is directly or indirectly involved in pathogenesis of numerous diseases (Djordjevic 2004) including inner ear disorders such as age-related hearing loss (Huang et al. 2000; Staecker et al. 2001) or acoustic trauma (Fetoni et al. 2013). Oxidative insult activates diverse intracellular stress signaling pathways, including members of mitogen-activated protein kinases (MAPK) family, tumor protein 53, AKT/PKB pathway, NF-κb, Janus kinases (JAK), and others (Martindale and Holbrook 2002). Some of them are associated with cell survival, while the others with cell death. One of the cell death types is apoptosis, initiated by several proapoptotic factors including p-38 MAPK and executive caspase-3.

The present study is focused on hair cells, which are sensory receptors responsible for signal transduction in the inner ear of vertebrates. Hair cells are very sensitive to oxidative stress and apoptosis seems to be accountable for removing damaged mechanosensory cells from the cochlea (Huang et al. 2000). There are many types of insult inducing oxidative stress in hair cells resulting in apoptosis and ototoxicity. They include heavy metals (Olivari et al. 2008), aminoglycosides (Ylikoski et al. 2002; Jiang et al. 2005), cisplatin (Alam et al. 2000), as well as noise (Henderson et al. 2006). Another ubiquitous ototoxic initiator is hydrogen peroxide (H_2_O_2_). H_2_O_2_ differs from the mentioned factors. The difference is that the involvement of heavy metals, aminoglycosides, cisplatin, or noise leads to ROS formation, whereas H_2_O_2_ is a ROS itself. Moreover, H_2_O_2_ unlike previous agents is an important endogenously produced molecule functioning under physiological conditions (Halliwell and Gutteridge 1990; Dehne et al. 2000). Reports from the last two decades assume the undeniable impact of free radicals on age-related hearing loss (McFadden et al. 2001; Seidman et al. 2002; Seidman and Vivek 2004; Jiang et al. 2007; Huang and Tang 2010; Someya et al. 2010). The intervention strategy against oxidative stress-induced ototoxicity could be the administration of antioxidant or antiapoptotic drugs (Seidman and Vivek 2004).

One of the antiapoptotic and antioxidant factors receiving much attention lately is the 38-amino acid form of pituitary adenylate cyclase-activating polypeptide (PACAP-38). PACAP-38 is a neuropeptide widely distributed in the nervous system and peripheral organs acting through three distinct receptors: PAC1-R, VPAC1-R, and VPAC2-R (Vaudry et al. 2009). Its sequence is evolutionarily well conserved, proving that PACAP-38 plays a role in important biological pathways (Arimura and Shioda 1995; Okazaki et al. 1995; Zhou et al. 2002). One of the studied PACAP-38 functions is its protective role against oxidative stress and oxidative stress damages. In vitro, it has been already revealed that PACAP-38 protects neural cells like cerebellar granule neurons (Vaudry et al. 2002), and non-neural cells including cardiomyocytes (Gasz et al. 2006), endothelial cells (Racz et al. 2007a, b) as well as hair cells (Racz et al. 2010). We have also shown that PAC1-R and the calcium-binding proteins in the inner ear are differently expressed in PACAP-deficient mice and in wild-type animals and that both types of mice respond differently to kanamycin-induced stress (Tamas et al. 2012; Nemeth et al. 2014). Whether PACAP-38 accomplishes protection against oxidative stress in the inner ear in vivo has not been yet investigated.

For this purpose, the zebrafish (*Danio rerio*) was used in the present study. Valuable biological characteristics make the zebrafish an excellent animal research model partially replacing the mammalian models. The zebrafish inner ear and lateral line consist of individual sense organs—neuromasts composed of hair cells, which resemble hair cells of mammalian inner ear (Dambly-Chaudiere et al. 2003). Moreover, the zebrafish lateral line is a favorite model system for investigating hair cells because of its superficial localization and conservative pattern making histological labeling and visualization quite easy. The lateral line with completely formed neuromasts appears at 24–30 h postfertilization (hpf), while the whole morphogenesis is rather completed at 3-day postfertilization (dpf) (Kimmel et al. 1995). Therefore, it is possible and relevant to use the larvae for various kinds of studies.

The aim of the present study was to demonstrate for the first time the effects of H_2_O_2_-induced oxidative stress in zebrafish neuromasts and to examine protective properties of PACAP-38 under oxidative stress conditions in the hair cells in vivo. Furthermore, we aimed at investigating whether the protective effect of the peptide is accomplished by reducing the level of active caspase-3 and involvement of p-38 MAPK pathway. The protective role of PACAP-38 investigated at cellular level was also validated by behavioral analyses.

## Materials and Methods

### Animals

In the study, 5 dpf Tubingen and Turku zebrafish strain was used. Adult fish were maintained at 28 °C with a 14-h light:10-h dark photoperiod and fed three times daily ad libitum with dry food and *Artemia* sp. *naupli*. The males and females were kept together in tanks. Spawning was made by transferring one male and one female to breeding tanks in the evening. The eggs were collected next morning and transferred into Petri dishes with embryo solution (50 eggs per 50 ml of embryo solution). Embryo solution (E3 medium) is a breeding medium containing 5 mM NaCl, 0.17 mM KCl, 0.33 mM CaCl_2_, and 0.33 mM MgSO_4_. The embryos were kept in the incubator at the 28.5 °C and 14 h light:10 h dark photoperiod without feeding until 5 dpf.

### Experimental Conditions

**Fig. 1:**
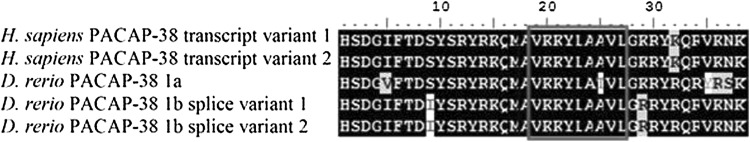
Juxtaposition of the human and zebrafish PACAP-38 amino acid sequence. The differences occur in eight amino acids in the whole PACAP-38 amino acid sequence. Human PACAP-38 receptor-binding site (*red frame*) differs from the zebrafish PACAP-38 isoform 1 (1a) in one amino acid, while the zebrafish PACAP-38 isoform 2 (1b) is 100 % homologous to that of human. *Source* http://www.ncbi.nlm.nih.gov/ (Color figure online)

The 5 dpf larvae were randomly divided into experimental groups. Each experimental group consisted of 15 individuals. The control group was incubated in embryo solution (E3 medium), the same which was used for breeding. To induce the oxidative stress resulting in caspase-3 activation in hair cells, the larvae were incubated in H_2_O_2_ solution (Hydrogen peroxide 30 % pure P.A.—basic, POCH BASIC, Cat No. BA5193111). Firstly, the exposure time and H_2_O_2_ dose were selected after several experiments aimed to create the most proper conditions for caspase-3 activation. The establishment of the most effective H_2_O_2_ concentration was achieved by dose-dependent manner study. Animals were divided into five H_2_O_2_ exposure groups: 0.1, 0.5, 1.5, 2.5, and 5 mM. Exposures lasted 1 h. For the purpose of our study, the 1.5 mM H_2_O_2_ was chosen (see Sect. Results). PACAP-38 was synthesized as previously described (Gasz et al. 2006). The sequence of PACAP-38 used in our study refers to mammalian PACAP-38, but using Bioedit Sequence Alignment Editor (BioEdit 7.0, Ibis Biosciences, Carlsbad, CA 92008) we demonstrated 80 % homology between the zebrafish and human peptide sequence (Fig. 1). In turn, the zebrafish receptor-binding site sequence corresponds to that of human in almost 100 % (Fig. 1). To investigate the ameliorative role of PACAP-38, the larvae were incubated for 1 h with a mixture of 1.5 mM H_2_O_2_ and 100 nM PACAP-38, following 1 h preincubation with 100 nM PACAP-38 only. 100 nM PACAP-38 dose was used based on previous studies, where 100 nM PACAP-38 was proven to be the most effective concentration to prevent from apoptosis several cell types, including culture of hair cells (Somogyvari-Vigh and Reglodi 2004; Racz et al. 2007a, b, 2010; Reglodi et al. 2011, 2015; Brown et al. 2014). The evaluation of H_2_O_2_ influence on p-38 MAPK phosphorylation was done by the incubation of the larvae in 1.5 mM H_2_O_2_ for different time periods: 5, 15, and 30 min to determine the minimum time point sufficient for p-38 MAPK activation in hair cells body. To examine PACAP-38 inhibitory effect in hair cells, the larvae were incubated for 15 min with a mixture of 1.5 mM H_2_O_2_ and 100 nM PACAP-38, following 1 h preincubation with 100 nM PACAP-38 only. Both, PACAP-38 and H_2_O_2_ were diluted from their stocks in E3 medium to ensure the individuals suitable and required for living conditions during the experiment. Each experiment was performed in 6-well plates. Each well represented an individual experimental condition: control, 1.5 mM H_2_O_2_, 100 nM PACAP-38 + 1.5 mM H_2_O_2_, and 100 nM PACAP-38. In each well, there were 15 larvae. This number is proper for possessing enough large groups without stressing the individuals. The experiments were carried out during daytime. To provide the standard maintenance conditions, 5 dpf larvae were set in the incubator with 28.5 °C. Plates were in all experiments protected from light by covering the well plates with aluminum foil to avoid H_2_O_2_ decomposition to water and oxygen.

### Whole-mount Immunohistochemistry

Immunohistochemistry was performed in both experiments. The zebrafish neuromasts are located superficially, therefore, whole-mount immunohistochemistry was sufficient to obtain the distinct and specific staining.

The 5 dpf larvae immediately after the experimental treatment were fixed with 4 % PFA over night (o/n) at 4 °C. Next, 4 % PFA was replaced with phosphate-buffered saline (PBS; pH 7.4) for 3 h and kept at 4 °C. After PBS prerinsing, the specimens were washed three times for 30 min in PBS with 0.3 % Triton X-100, (PBST; pH 7.4) on slow shaking. After this time, all the groups were set in preincubation buffer containing PBST with 1 % dimethyl sulfoxide (DMSO), 4 % normal goat serum (NGS), and 0.1 sodium azide (blocking solution) o/n at 4 °C. Subsequently, the specimens were incubated with primary antibodies.

To visualize the neuromasts, an antiacetylated α-tubulin antibody was used. To label the apoptotic cells, an antiactive caspase-3 antibody was used. The activation of p-38 MAPK in the hair cells was detected using an antiphospho-p-38 antibody (Antibodies are listed in Table 1). The primary antibodies were diluted in a blocking solution. The incubation with the primary antibodies was o/n at 4 °C on slow shaking. Afterwards, the samples were washed carefully 3 × 30 min in PBST at room temperature and incubated with the secondary antibodies (Antibodies are listed in Table 1) diluted in a blocking solution and incubated o/n at 4 °C on slow shaking. Next day, all the individuals were intensively washed 3 × 30 min in PBST, next 1 × 60 min in 50 % glycerol in PBS, then mounted in 80 % glycerol in PBS.

**Table 1:** Primary and secondary antibodies

Primary antibodies						
Antigen	Immunogen	Host	Clonality	Dilution	Company	Catalog no
Phospho-p-38 MAPK (Thr180/Tyr182)	A synthetic phosphopeptide corresponding to residues surrounding Thr180/Tyr182 of human p-38 MAPK	Rabbit	Monoclonal	1:500	Cell signaling technology	9215
Cleaved caspase-3 (Asp175)	A synthetic peptide corresponding to amino-terminal residues adjacent to (Asp175) in human caspase-3	Rabbit	Polyclonal	1:500	Cell signaling technology	9661
Acetylated tubulin	Acetylated alpha-tubulin from the axoneme of sea urchin sperm flagella	Mouse	Monoclonal	1:1000	Sigma	T6793

Secondary antibodies						
Antigen	Fluorophore	Host	Dilution	Company	Catalog no	
Mouse IgG	Alexa 488	Goat	1:1000	Invitrogen	A-11029	
Rabbit IgG	Alexa 568	Goat	1:1000	Invitrogen	A-11036	
Rabbit IgG	Alexa 555	Goat	1:1000	Invitrogen	A-21431	

### Microscopy and Quantification

The visualization of active caspase-3 staining was accomplished using LSM 700 confocal laser scanning microscope (Zeiss, Germany). To obtain the desirable image ×20 and ×40 objectives were used. Stacks of images were composed into one to obtain maximum intensity projection images with ZEN 2009 software (Zeiss, Germany). The quantification was made by counting hair cells visibly affected by active caspase-3 on the images using z-stack. Z-stack tool enabled quantifying every single hair cell noting its 3D structure.

The visualization of phospho-p-38 MAPK was achieved using Leica TCS SP2 AOBS confocal microscopy system (Leica Microsystems, Mannheim, Germany). To obtain the desirable image, ×40 objectives and 4.0 digital magnifications were used. Like previously, stacks of images were composed into one to obtain maximum intensity projection images with Leica Confocal software (Leica Microsystems). The morphology of phospho-p-38 staining was different than that of caspase-3 and the cell borders were hardly distinguishable; therefore, in this experiment, more relevant measurement technique was based on determining the volume (µm^3^) of phosphorylated form of p-38 MAPK in the whole neuromast. For this purpose, the Imaris software (Bitplane AG, Switzerland) was used. The measurement threshold was determined by selecting the best value which labeled only specific stain in the neuromast body, not the background. The most specific threshold value was 30 units. Using the set threshold, every single neuromast was analyzed and the value of phosphorylated p-38 volume was generated by Imaris software in cubic micrometers (µm^3^).

### Behavioral Analyses

Analyses of basic movement parameters (described according to Zhou et al. 2014 in Table 2) were performed to reveal the influence of PACAP-38 on behavior under oxidative stress condition. For this purpose, two kinds of experiments were performed. In the first one, the 1.5 mM H_2_O_2_ exposure lasted 1 h while in the second one, exposure lasted 15 min. In both cases, the preincubation with 100 nM PACAP-38 was 1 h. Additionally, both experiments involved the control group (E3 medium) and the group consisting of the larvae incubated in 100 nM PACAP-38 for 1 h to check whether PACAP-38 has any influence on the behavior. Each experiment was repeated three times using the same behavioral tracking system. After exposure time, each solution used in the experiment was replaced with E3 medium. The behavioral procedure was applied according to Cario et al. (2011) and done independently as described. Using a Pasteur pipette, the larvae were transferred with some E3 medium into individual wells of the special 48-well plate, where wells are separated by black areas and have optical glass bottoms. After loading the larvae in the well plate, each well was filled with fresh E3 medium to obtain slightly convex meniscus at the top and video recordings were started. We followed the videography described by Zhou et al. (2014). For our purpose, the upright videography was modified and applied. The camera was positioned beneath the 48-well plate, which was transilluminated with a white light from above. This construction enabled the recording through the optical glass bottom of the 48-well plate instead of the buffer convex meniscus. The experiment was protected from external stimuli, by running it in a specially adapted room, with the temperature maintained at 26 °C, without any extra light or sound sources. 50 min videos were recorded with Miotic Image plus 2.0 adjusted manually, to get the best video image quality. The analysis and quantification of 5 dpf larvae motor function was conducted using open source Matlab applications *LSRtrack* and *LSRanalyze* kindly provided by Professor Edward A. Burton, University of Pittsburgh School of Medicine, USA. Each parameter in the threshold panel was set manually to optimize tracking accuracy and get the most reliable tracking analyses. The analysis of tracked videos was based on centroid tracking, which includes the main behavioral parameters like mean velocity, active velocity, and % time moving (described in Table 2).

**Table 2:** Summary data outputs of behavioral parameters generated by *LSRanalyze*

Name	Definition
Mean velocity	*V*_M_ = total larval centroid displacement (mm)/duration of recording (s)
Active velocity	*V*_A_ = *V*_M_/*T* % (an approximation of velocity during movement events)
% Time moving	*T* % = number of frame transitions showing larval centroid displacement/number of frames in recording

### Statistical Analyses

The statistical analyses were performed using GraphPad Prism, version 5.0. Each analysis concerning data with Gaussian assumption was made using one-way ANOVA test, One-way analysis of variance with Tukey multiple comparisons tests as a posttest. Data analyses not assuming Gaussian distribution were based on one-way ANOVA test, Kruskal–Wallis test with Dunn’s multiple comparisons test as a posttest. The error bars are reported as mean ± SEM of the mean. Significance level was set at *α* = 0.05 (95 % confidence intervals).

## Results

### H_2_O_2_-Induced Hair Cell Death in Dose-Dependent Manner

**Fig. 2:**
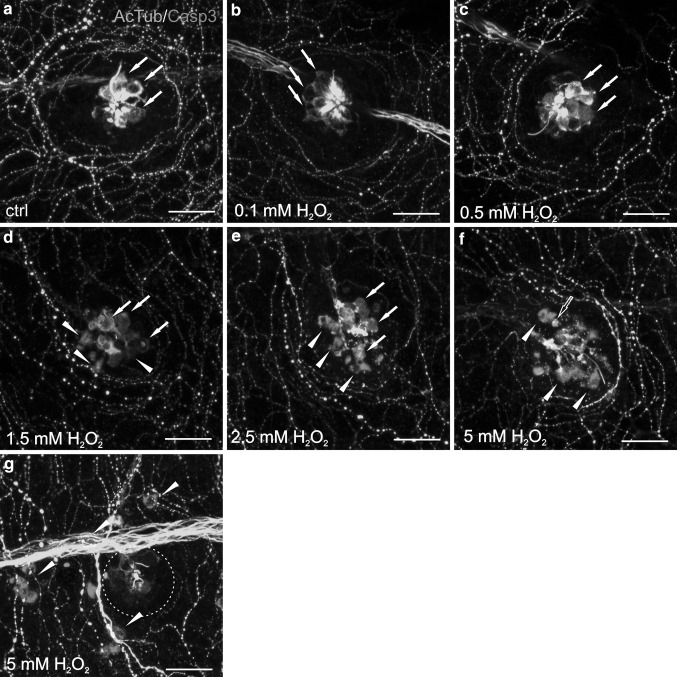
Morphology of L2 neuromast hair cells in 5 dpf zebrafish (see Fig. 3) exposed for 1 h to H_2_O_2_ in dose-dependent manner, double-stained with antibodies against acetylated α-tubulin (AcTub) (*green*) and caspase-3 (Casp3) (*magenta*). The visualization was accomplished using a Zeiss LSM-700 confocal microscope. Life hair cells were stained with anti-AcTub antibody (*arrows*) and the apoptotic hair cells were marked with anti-Casp3 antibody (*arrow heads*). **a** Hair cells in the control group characterized by proper morphology without any apoptosis. **b**, **c** Low H_2_O_2_ doses resulted in unchanged hair cells morphology after both 0.1 mM **(b)** and 0.5 mM **(c)** H_2_O_2_ exposure. **d** 1.5 mM is the minimum H_2_O_2_ concentration resulted in strong and evident apoptosis recognized based on Casp3 detection (*arrow heads*). Casp3 immunoreactive (IR) hair cells undergoing apoptosis are irregularly shaped and fragmented, detaching from neuromast rosette. **e** neuromast rosette is more violated and blebs are observed occasionally. **f** Hair cells are entirely destroyed and those remaining ones are only Casp3 IR. Some separated, shrunken cells are observed (*hollow arrow*) pointing to advanced apoptosis events. In some cases, 5 mM H_2_O_2_ caused neuromast destruction without Casp3 appearance, suggesting that other death pathways may be involved as well (*dotted circle*) **(g)**. The only centrally located rosette remaining is neuromast innervation **(f**, **g)**. In 1.5 mM **(d)** and 2.5 mM **(e),** H_2_O_2_ concentration apoptosis is restricted to the neuromast hair cells, while 5 mM H_2_O_2_ exposure leads to other cell types death, e.g., skin cells **(g)**. *N* = 15/group. *Scale bars* 20 µm (Color figure online)

To demonstrate the H_2_O_2_ dose-dependent manner toxicity toward the neuromasts, following H_2_O_2_ concentrations were chosen: 0.1, 0.5, 1.5, 2.5, and 5 mM (Fig. 2). Neuromasts in the control group were characterized by normal-shaped hair cells without any apoptotic events (Fig. 2a). Similar observations were made in groups exposed to 0.1 and 0.5 mM H_2_O_2_, where no apoptosis was determined (Fig. 2b, c). The minimum H_2_O_2_ concentration which resulted in strong, evident apoptosis estimated based on caspase-3 (Casp3) appearance was 1.5 mM (Fig. 2d). The shape and the integrity of cells were normal; however, the presence of curled kinocilia was reported, while in the control and 0.1 and 0.5 mM exposed animals they remained straight. Moreover, some hair cells detached from neuromast rosette, appearing irregular and fragmented, clearly pointing to apoptosis. The exposure to 2.5 mM H_2_O_2_ resulted in the amount of caspase-3 immunoreactive (IR) hair cells within the neuromast comparable with 1.5 mM exposure, however the hair cells rosette remained much more disintegrated, resulting in higher amount of hair cells occurring outside the neuromast (Fig. 2e). Additionally, some blebs characterized by the decoupling of the cytoskeleton from the plasma membrane were reported. Our aim was to investigate the H_2_O_2_ concentration resulting in apoptosis strictly localized within the neuromast. Exposure to 1.5 and 2.5 mM H_2_O_2_ concentration met these criteria proving that the model is appropriate. In 5 mM H_2_O_2_ concentration, much more advanced apoptosis was observed (Fig. 2f). The hair cells rosette was totally destroyed and almost each cell was separated from each other, as well as characteristic single shrunken hair cells were recognizable. Remarkably, hair cells were characterized by strong fragmentation and cells without caspase-3 were hardly detectable. Occasionally, in single-destroyed neuromasts caspase-3 was not apparent, suggesting that they are not in apoptosis anymore or followed another death pathway (Fig. 2g). Furthermore, apoptotic events appeared within other cell types, suggesting that 5 mM H_2_O_2_ concentration is too strong and not specific for affecting only the neuromast hair cells (Fig. 2g). It should be noticed that in 1.5, 2.5, and 5 mM H_2_O_2_-exposed groups, the acetylated α-tubulin (AcTub) and caspase-3 (Casp3) colocalization is hardly detectable, pointing to the caspase-3 properties in cleaving cytoskeletal proteins including α-tubulin. Based on this experiment, the most effective H_2_O_2_ concentration was 1.5 mM, therefore it was used for the purpose of the present study.

### The Effect of PACAP-38 on H_2_O_2_-Induced Hair Cell Death

**Fig. 3:**
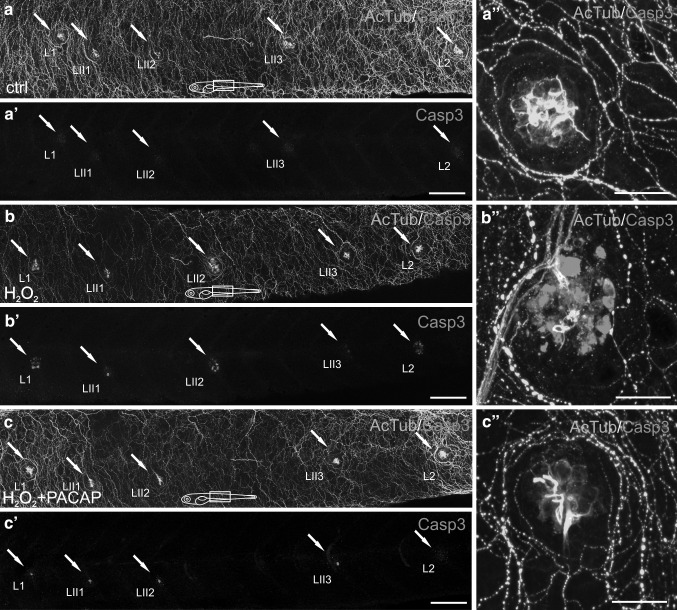
Immunohistochemical staining of the 5 dpf zebrafish neuromasts using antibodies against acetylated α-tubulin (AcTub) (*green*) and caspase-3 (Casp3) (*magenta*). The visualization was accomplished using a Zeiss LSM-700 confocal microscope. The hair cells were stained with AcTub antibody and the apoptotic hair cells were marked with Casp3 antibody. **a**, **b**, **c** five trunk neuromasts (*arrows* from *left* to *right*: L1, LII1, LII2, LII3, L2, respectively) in the control animals (**a**), in the animals after 1 h 1.5 mM H_2_O_2_ exposure (**b**) and in the animals after 1 h 1.5 mM H_2_O_2_ + 100 nM PACAP-38 exposure preceded by 1 h preincubation with 100 nM PACAP-38 only (**c**) presented in a merged form with marked AcTub and Casp3 double-staining. **a’**–**c’** Casp3 in hair cells of neuromasts L1, L II1, LII2, LII3, L2 (*arrows*) in the control animals (**a’**), in 1 h 1.5 mM H_2_O_2_-exposed group (**b’**) and in 1 h 1.5 mM H_2_O_2_ + 100 nM PACAP exposure + 1 h 100 nM PACAP preincubation group (**c’**) visualized without AcTub. **a”**–**c”** the presence of the apoptotic hair cells in the trunk neuromast L2 in the control animals (**a”**), in the animals after 1 h 1.5 mM H_2_O_2_ exposure (**b”**) and in the animals after 1 h 1.5 mM H_2_O_2_ + 100 nM PACAP exposure preceded by 1 h preincubation with 100 nM PACAP only (**c”**). The immunohistochemical staining is visualized in a merged form, where the AcTub and Casp3 double staining is distinctly marked. *N* = 15/group. *Scale bars* a, a’; b, b’; c, c’ 100 µm; a”; b”; c” 20 µm (Color figure online)

**Fig. 4:**
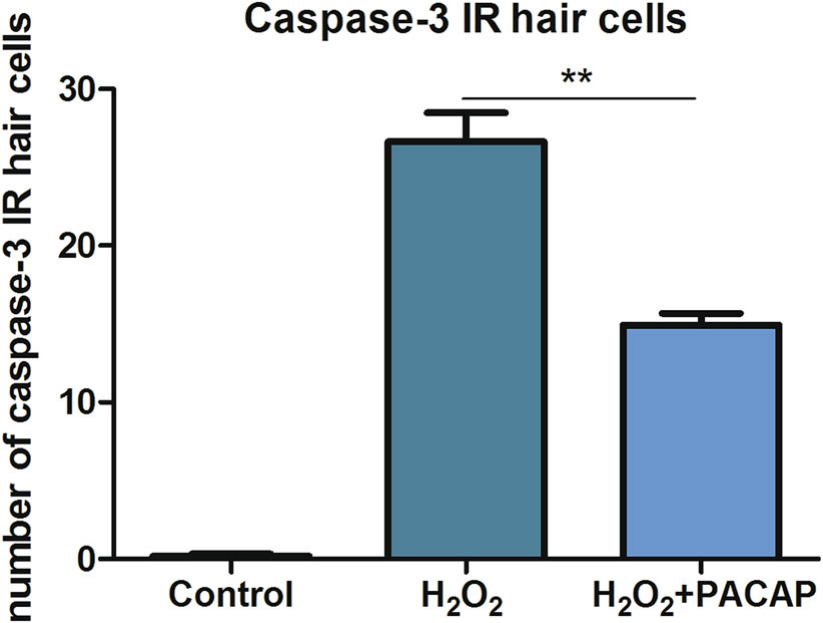
The influence of PACAP-38 on the number of the apoptotic hair cells marked with active caspase-3 after 1 h 1.5 mM H_2_O_2_ exposure. PACAP-38 preincubation resulted in a significant decrease in the number of the apoptotic hair cells in PACAP-38-treated group as compared to that determined in the H_2_O_2_-treated group (one-way ANOVA, Kruskal–Wallis test with Dunn’s posttest, GraphPad Prism 5, *p* < 0.01). Quantification was performed by counting caspase-3 immunoreactive (IR) hair cells in each trunk neuromast under a confocal microscope. *N* = 15/group

To evaluate the protective influence of PACAP-38 on H_2_O_2_-induced hair cell death, the appropriate dose and incubation time for H_2_O_2_ had to be established. The best H_2_O_2_ concentration which evoked the caspase-3 activation was 1.5 mM. The minimum exposure time resulted in caspase-3 IR hair cells was 1 h; 15 min—the other exposure time point used in our study was found to be insufficient to trigger caspase-3 activation (data not shown). These two experimentally established parameters were combined and used in the subsequent study. 1 h 5 dpf zebrafish exposition to 1.5 mM H_2_O_2_ resulted in a marked increase in the number of caspase-3 IR hair cells in comparison to that found in the control group. 5 dpf larvae coincubation with 1.5 mM H_2_O_2_ and 100 nM PACAP-38 following 1 h preincubation with 100 nM PACAP-38 alone resulted in a nearly two-fold decrease in the number of caspase-3 IR hair cells in PACAP-38-treated group in comparison to H_2_O_2_-exposed group (Figs. 3, 4).

### The Effect of PACAP-38 on the Volume of Phosphorylated p-38 MAPK in Hair Cells

**Fig. 5:**
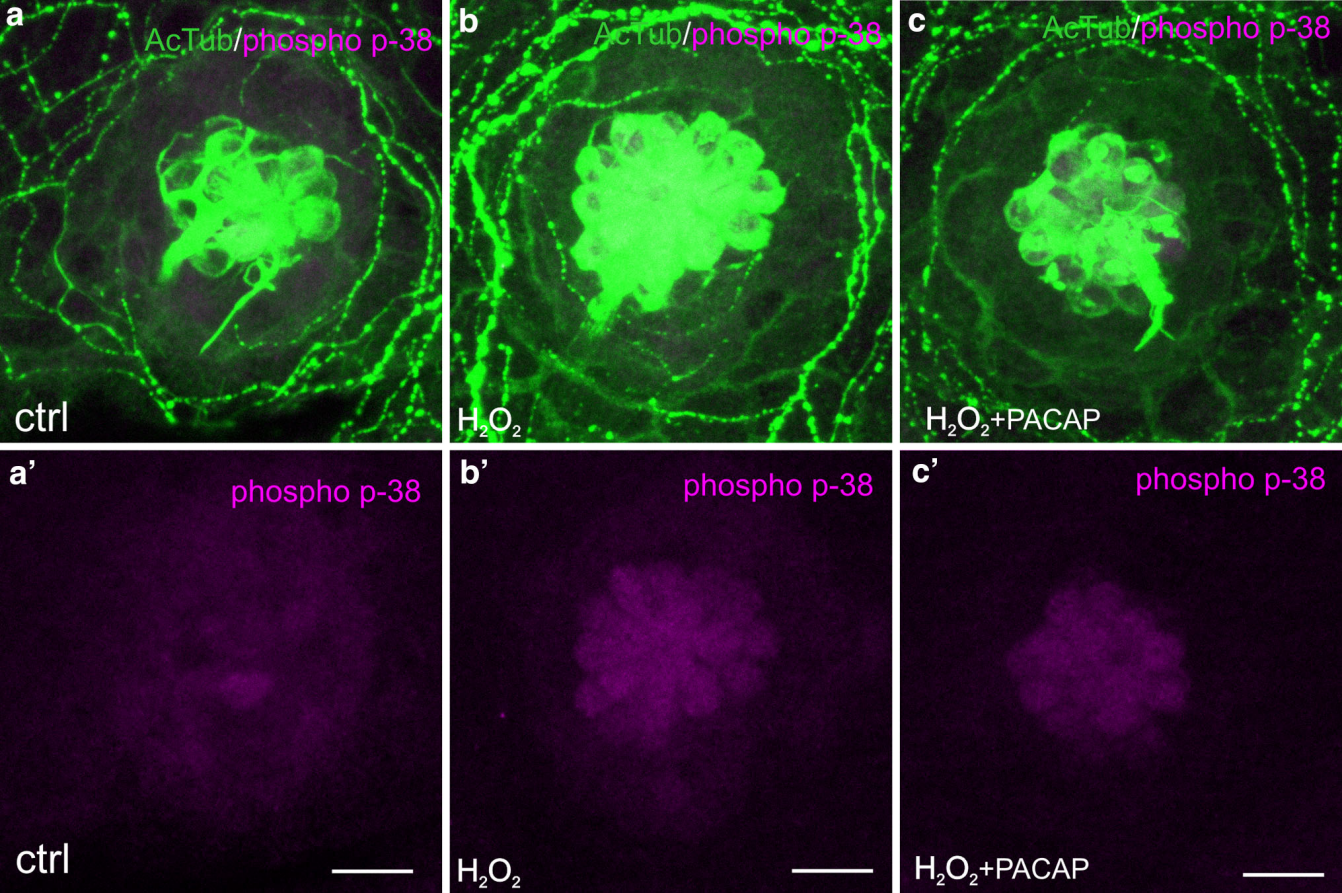
Immunohistochemical staining of the zebrafish neuromasts using antibodies against acetylated α-tubulin (AcTub; *green*) and phosphorylated p-38 MAPK (phospho-p-38 MAPK; *magenta*). The visualization was achieved using a confocal microscope. **a**–**c** the trunk neuromast L2 with the hair cells affected with phosphorylated form of p-38 MAPK presented in a merged form with marked AcTub and phospho-p-38 MAPK double-staining. **a’**–**c’** the presence of phophorylated form of p-38 MAPK in the hair cells. Single hair cells with activated p-38 were found in the control individuals (**a**, **a’**). 1.5 mM H_2_O_2_ exposure resulted in an unequivocal increase in the volume (µm^3^) of phospho-p-38 MAPK in the hair cells (**b**, **b’**). 100 nM PACAP-38 1 h preincubation did not change the volume of phospho-p-38 MAPK in the hair cells as compared to that determined in the 1.5 mM H_2_O_2_-treated group (**c**, **c’**). *N* = 15/group. *Scale bars* 10 µm (Color figure online)

**Fig. 6:**
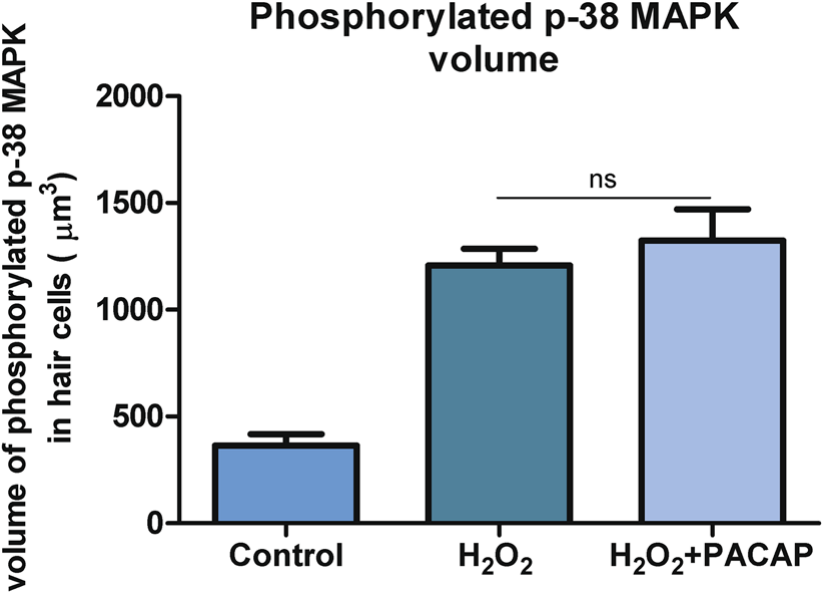
The influence of PACAP-38 on the volume (µm^3^) of phosphorylated form of p-38 MAPK in the hair cells after 15 min 1.5 mM H_2_O_2_ exposure. 1.5 mM H_2_O_2_ exposure resulted in a nearly three-fold increase in phospho-p-38 MAPK volume in the hair cells. 100 nM PACAP-38 preincubation did not significantly change the volume of phosphorylated form of p-38 MAPK in the hair cells in comparison to that found in the 1.5 mM H_2_O_2_-treated group (one-way ANOVA, Kruskal–Wallis test with Dunn’s posttest, GraphPad Prism 5, *p* > 0.05). Quantification was performed using Imaris software. Detected phospho-p-38 MAPK was quantified as volume of phospho-p-38 MAPK in the hair cells. *N* = 15/group

The connection between p-38 MAPK pathway and reactive oxygen species has been clearly established by others (Zhuang et al. 2000; Wang et al. 2003; Ito et al. 2006). To examine whether H_2_O_2_ stimulates p-38 MAPK, 5 dpf zebrafish were incubated with 1.5 mM H_2_O_2_ for 5, 15, and 30 min. P-38 MAPK phosphorylation in the hair cells occurred already after 5 min and reached the maximum level after 30 min exposure (data not shown). Exposure to 1.5 mM H_2_O_2_ for 15 min resulted in nearly three-fold increase in phospho-p-38 MAPK volume in the hair cells compared to the control value (Figs. 5a, a’, b, b’, 6). It should be noted that single p-38 MAPK IR hair cells were found also in the control larvae (Figs. 5a, a’, 6). To evaluate the inhibitory effect of PACAP-38 on p-38 MAPK phosphorylation, the larvae were incubated in 1.5 mM H_2_O_2_ for 15 min, following 100 nM PACAP-38 preincubation. No ameliorative effect of PACAP-38 on phospho-p-38 MAPK volume in hair cells was observed (Figs. 5c, c’, 6).

### Behavior

**Fig. 7:**
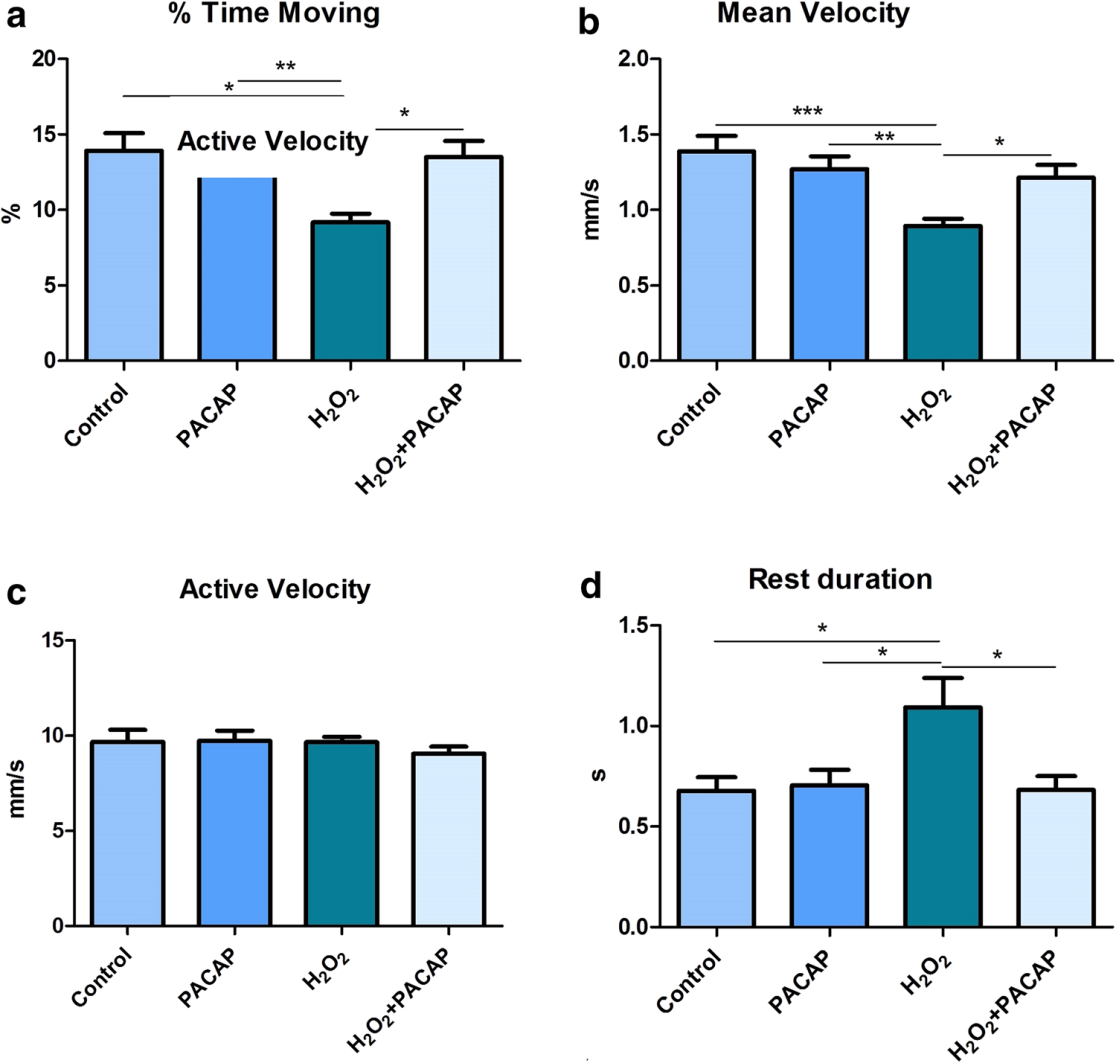
Effects of PACAP-38 preincubation on behavioral parameters of 5 dpf larvae after 15 min 1.5 mM H_2_O_2_ exposure. **a**, **b**, **d**: results obtained in 100 nM PACAP-38 + 1.5 mM H_2_O_2_-treated group were statistically significantly different from those obtained in the 1.5 mM H_2_O_2_ group (one-way ANOVA, One-way analysis of variance with Tukey posttest, GraphPad Prism 5, *p* < 0.05). PACAP-38 resulted in a statistically significant contemporaneous increase in the % time moving (**a**) and the mean velocity (mm/s) (**b**) and a decrease in the rest duration (s) (**d**). **c** the active velocity as an outcome of MV/%TM formula was not statistically significantly different between all the experimental groups (one-way ANOVA, One-way analysis of variance with Tukey posttest, GraphPad Prism 5, *p* > 0.05). *N* = 12/group

**Fig. 8:**
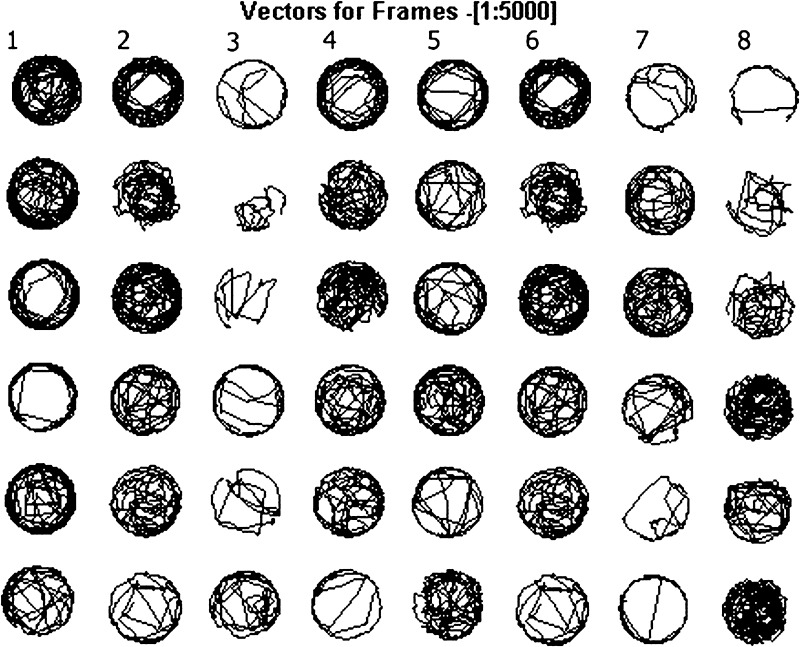
Vector plot showing swimming paths of zebrafish in the representative well-plate with 48 zebrafish larvae during 50-min recording. Swimming paths of the control (columns* 1* and* 5*), 100 nM PACAP-38 (columns* 2* and* 6*), and 1.5 mM H_2_O_2_ + 100 nM PACAP-38-treated group (columns* 4* and* 8*) presented higher density than those observed in the 1.5 mM H_2_O_2_-exposed group (columns* 3* and* 7*). Individual groups were distributed among the well-plate randomly to avoid the circumstantial influence of external factors on one of the group disposed in a particular part of the 48-well plate

Zebrafish neuromasts as mechanoreceptors are, inter alia, responsible for detection of water movements, thus they influence the behavior and movement parameters. Their dysfunction can strongly affect behavioral traits. A 50 min tracking of 5 dpf larval fish indicated that PACAP-38 has an ameliorative influence on basic movement behavior impaired by H_2_O_2_. Firstly, any effect of PACAP-38 on the behavior was excluded. There was no statistically significant difference in all investigated parameters between the control group (% time moving $\bar{x}$ = 13.92 %; *SE* = 1.17; mean velocity = 1.38 mm/s; *SE* = 0.1, active velocity $\bar{x}$ = 9.68 mm/s; *SE* = 0.62, rest duration $\bar{x}$ = 0.67 s; *SE* = 0.06) and the group incubated with 100 nM PACAP-38 for 1 h (% time moving $\bar{x}$ = 15.24 %; *SE* = 1.27, mean velocity $\bar{x}$ = 1.27 mm/s; *SE* = 0.08, active velocity $\bar{x}$ = 9.72 mm/s; *SE* = 0.52, rest duration $\bar{x}$ = 0.7 s; *SE* = 0.07) (Fig. 7a–d). 1 h 1.5 mM H_2_O_2_ exposure significantly declined the measured behavioral parameters in comparison to the control values. The preincubation of the 1.5 mM H_2_O_2_-exposed group with 100 nM PACAP-38 for 1 h did not significantly influence the locomotor parameters, however, there was a visible trend pointing to PACAP-38 efficacy (data not shown). Likewise, exposure to 1.5 mM H_2_O_2_ for 15 min significantly decreased all studied behavioral parameters as compared to control levels. In this case, 100 nM PACAP-38 1 h preincubation was sufficient to improve the investigated behavioral elements impaired by H_2_O_2_. First, PACAP-38 treatment resulted in increased percentage of movement events. The time of larval centroid displacement in H_2_O_2_-exposed group preincubated with PACAP-38 was about one-third longer ($\bar{x}$ = 13.50 %; *SE* = 1.08) than that observed in H_2_O_2_-exposed group ($\bar{x}$ = 9.17 %; *SE* = 0.58) and did not differ from that determined in the control group ($\bar{x}$ = 13.92 %; *SE* = 1.17) (Fig. 7a). Another parameter ameliorated by PACAP-38 was the mean velocity (mm/s) of the larval movement. There were no statistical differences between the control group ($\bar{x}$ = 1.38 mm/s; *SE* = 0.1) and H_2_O_2_-exposed group preincubated with 100 nM PACAP-38 ($\bar{x}$ = 1.22 mm/s; *SE* = 0.08). Compared to H_2_O_2_-exposed group ($\bar{x}$ = 0.8 mm/s; *SE* = 0.04), PACAP-38 preincubation contributed to one-third increase in swimming speed (Fig. 7b) ($\bar{x}$ = 1.22 mm/s; *SE* = 0.08). No differences in the active velocity (mm/s) between all the groups were observed (control $\bar{x}$ = 9.68 mm/s; *SE* = 0.62; PACAP-38 $\bar{x}$ = 9.72 mm/s; *SE* = 0.52; H_2_O_2_$\bar{x}$ = 9.65 mm/s; *SE* = 0.28; H_2_O_2_ + PACAP-38 $\bar{x}$ = 9.06 mm/s; *SE* = 0.37) (Fig. 7c). This is due to the formula describing this parameter, where the active velocity is in a direct proportion to the mean velocity and in an inverse proportion to the % time moving. Additionally, the preincubation of H_2_O_2_-exposed group with PACAP-38 resulted in a diminished larvae rest duration (s) (duration of contiguous video frames showing no displacement of larval centroid) ($\bar{x}$ = 0.68 s; *SE* = 0.06) in relation to the H_2_O_2_-exposed group ($\bar{x}$ = 1.09 s; *SE* = 0.14) demonstrating similar values to the control ($\bar{x}$ = 0.67 s; *SE* = 0.06) (Fig. 7d). Behavioral impairments are presented in graphical form revealing disturbed swimming paths of zebrafish larvae in H_2_O_2_ exposed group (Fig. 8).

## Discussion

Reactive oxygen species underlie many injuries and critical diseases, including age-related hearing loss (Darrat et al. 2007), whose prevalence is expected to rise among aging population (Gratton and Vazquez 2003; Gates and Mills 2005; Yamasoba et al. 2007; Roth et al. 2011). Hair cells of the organ of Corti have been shown to demonstrate high susceptibility to free radical damage (Alam et al. 2000; Ylikoski et al. 2002; Jiang et al. 2005; Henderson et al. 2006; Olivari et al. 2008). Increased level of reactive oxygen species violates proper functioning of the hair cells, leading to the cytochrome c release from the mitochondria and caspase pathway activation (Rybak et al. 2007). In the absence of sufficient antioxidant, defense mechanisms, or without administration of antioxidant drugs caspases action triggers apoptotic cell death.

The present study has revealed that H_2_O_2_ leads to apoptotic events in the zebrafish hair cells through caspase-3 and p-38 MAPK activation. H_2_O_2_ dose-dependent manner study demonstrated that the minimum effective concentration of H_2_O_2_, during 1 h exposure, leading to caspase-3 activation is 1.5 mM. Higher concentrations resulted in greater neuromast disruptions, where in addition to the caspase-3 appearance, the whole neuromast morphology was abnormal. The range of the H_2_O_2_ concentration resulted in caspase-3 activation specifically and directly in the neuromast hair cells was quite small, because already at concentration of 5 mM, the apoptosis started to appear in other organs, especially within the skin of the trunk and head. Other studies confirm caspase-3 and p-38 MAPK involvement in hair cells oxidative damages (Wei et al. 2005; Sha et al. 2009; Tabuchi et al. 2010; Jamesdaniel et al. 2011; Park et al. 2012; Bas et al. 2012; Shin et al. 2014). However, most of these results have been obtained in in vitro studies (Wei et al. 2005; Sha et al. 2009; Park et al. 2012; Bas et al. 2012; Shin et al. 2014). In our research, we have used the zebrafish, whose neuromasts consisting of hair cells are an ideal tool for investigating the auditory system in vivo. Zebrafish hair cells are morphologically and functionally developed already at 27 hpf (Tanimoto et al. 2011), thus 5 dpf larvae provide a relevant model. The hair cells are characterized by strong tubulin immunoreactivity due to the presence of tubulin in the cytoplasm and the kinocilia. It has been demonstrated that oxidative stress results in the alteration of tubulin and disassembly of microtubules (Boonstra and Post 2004; Santa-Maria et al. 2005; Parker et al. 2014; Mackeh et al. 2014) and H_2_O_2_ causes their depolymerization (Lee et al. 2005). This could explain high sensitiveness of the hair cells to oxidative disturbances.

The aim of this study was to investigate the ameliorative influence of PACAP-38 on hair cells during oxidative stress. We analyzed both PACAP-38 action at the cellular level and the behavioral aspects of its accomplishment. PACAP-38 unveiled antiapoptotic properties through a marked reduction in caspase-3 activation upon H_2_O_2_ exposure. PACAP-38 antiapoptotic abilities have been previously shown in rat cerebellar granule neurons (Vaudry et al. 2002), rat cardiomyocytes (Gasz et al. 2006), mouse endothelial cells (Racz et al. 2007a, b), as well as chicken hair cells (Racz et al. 2010) in vitro. Similar effects were observed in PACAP deficient mice, which were more sensitive to oxidative damages than the wild-type animals (Vaudry et al. 2005; Horvath et al. 2010). Our study has demonstrated for the first time antioxidant properties of exogenously administered PACAP-38 in the hair cells in vivo. Incubation of 5 dpf zebrafish for 1 h in 100 nM PACAP-38 solution was enough to allow influx and passage of PACAP-38 across the integumentary system to the neuromasts. Peptides are easily taken up into the zebrafish embryo and larvae from the medium, a fact that has been demonstrated several times (Williams and Holder 2000; Donnini et al. 2010). In mammals, PACAP-38 acts via its specific receptors PAC1-R and VPAC1-R and VPAC2-R. The two receptors PAC1-R and PAC2-R, found in the zebrafish, present congruous binding specificity for both zebrafish and human PACAP-38, and for PACAP-27 (Wei et al. 1998), despite the fact that in the zebrafish two PACAP-38 forms—PACAP1 and PACAP2—exist (Wu et al. 2006). The localization of PACAP receptors and PACAP neuromodulatory functions in the auditory system has been investigated several times. Transcriptomic studies have revealed specific expression pattern of mRNA for five PAC1-R splice variants in microdissected cochlear subfractions (Abu-Hamdan et al. 2006). Further investigations have localized PACAP and PAC1-R in the co chlea, pointing to PACAPs role as an efferent neuromodulator altering cholinergic, dopaminergic and glutamatergic efferent modulation of hair cell afferent signaling (Drescher et al. 2006). We described altered expression of PAC1-R in PACAP-deficient mice cochlea compared to wild-type mice (Tamas et al. 2012). The modulatory character of PACAP has also been reported in the superior olive, which is considered to be a major complex involved in multiple aspects of hearing (Reuss et al. 2009).

Our study has revealed the involvement of PACAP-38 in protection of hair cells, mostly via the antiapoptotic pathway. In comparison to the H_2_O_2_-treated group, PACAP-38 treatment resulted in a nearly two-fold decrease in the number of caspase-3 IR hair cells upon H_2_O_2_ exposure. The impact of PACAP-38 on active caspase-3 reduction has been demonstrated several times in the cells of various origins (Vaudry et al. 2000, 2002; Gasz et al. 2006; Pirger et al. 2008; Racz et al. 2010). It has been suggested that PACAP-38 influences caspase-3 via protein kinase A (PKA; cAMP-dependent protein kinase) and protein kinase C (PKC) but not by ERK-type MAPK transduction pathways (Dejda et al. 2011; Bhandare et al. 2016). However, the question dealing with the influence of other upstream pathways has remained still open. Our study revealed that the PACAP-38 preincubation did not change the level of the phosphorylated form of p-38 MAPK which was increased after H_2_O_2_ exposure. The relationship between reactive oxygen species and p-38 MAPK has been well established earlier (Zhuang et al. 2000; Wang et al. 2003; Ito et al. 2006). MAPK pathway is moderated by different stress stimuli including ROS. P-38 MAPK activation by ROS is mostly associated with cell death and p-38 MAPK inhibitors lead to significant cell protection against oxidative stress (Matos et al. 2005; Yamada et al. 2012; Ki et al. 2013). On the other hand, there is some evidence that p-38 MAPK activation up-regulates antioxidant genes, such as *SOD*-*1* and *CAT*, promoting cell survival (Gutierrez-Uzquiza et al. 2011). PACAP-38 influences p-38 and the rest of the family members of the MAP kinases—ERK and JNK. In general, the properties of PACAP-38 are strictly antiapoptotic, because it increases ERK activity and suppresses JNK and p38 activity (Hashimoto et al. 2003; Li et al. 2004, 2006; Vlotides et al. 2004; Lee and Suk 2004; Kim et al. 2006; Harfi and Sariban 2006; El Zein et al. 2007). However, there are findings suggesting opposite PACAP-38 effects on p-38 MAPK resulting in its activation (Moroo et al. 1998; Sakai et al. 2002; May et al. 2010). This implies that effects of PACAP-38 on p-38 MAPK depend on experimental conditions and cell type, and need further investigations.

Behavioral analyses completed in the present study have revealed PACAP-38 ameliorative effect on the basic movement parameters, undoubtedly supporting the protective role of PACAP in hair cells against oxidative stress. Based on the H_2_O_2_ dose-dependent manner study, for present behavioral assay, the 1.5 mM H_2_O_2_ concentration was chosen. 1.5 mM H_2_O_2_ concentration is an appropriate and relevant model, restricted only to the neuromast hair cells, therefore, it can be assumed that the protective effect of PACAP is mostly directed toward neuromast hair cells. PACAP-38 administration after 1 h H_2_O_2_ exposure did not change behavior in a statistically significant way. This indicates that despite the active caspase-3 inhibition by PACAP-38, the neuromast functional properties can be affected by general ROS toxicity. ROS toxicity may also concern whole organisms, precluding larval normal behavior. However, the caspase-3 and phospho-p-38 MAPK immunostainings after 1.5 mM H_2_O_2_ exposure were specifically detectable only within the neuromast hair cells, emphasizing that the H_2_O_2_ dose applied was specifically selected. In turn, after shorter time of H_2_O_2_ exposure, PACAP-38 preincubation resulted in an improvement of investigated behavioral parameters, which corresponded with the control values. The lack of differences in behavior between PACAP-38 and the control group allows to hypothesize that any impact of PACAP-38 on the locomotor parameters results from its protective functions, not from inherent properties to increase the mobility. However, there is an evidence suggesting an arousal effect of PACAP-38 in zebrafish with PACAP-38 overexpression (Woods et al. 2014). In contrast to our findings, it was shown that exceeding physiological level of PACAP-38 contributed to a decrease in zebrafish rest duration (s) and increase in movement frequency (Hz) (Woods et al. 2014). The discrepancies between the present and the earlier observations may result from the way of PACAP-38 administration. In all probability, contrary to the results of the studies involving zebrafish with PACAP-38 constant overexpression, the exogenous PACAP-38 administration from E3 medium applied in our investigations did not affect the nervous system and did not change the behavior. Therefore, it can be assumed that the incubation of the larvae in the PACAP-38 solution was enough for the penetration of the peptide through the integument and affected the relatively externally localized neuromasts, but the PACAP-38 influence on nervous system is poorly feasible via the exogenous and time-limited approach. PACAP-38 produces a physiological response to oxidative stress, acting as an antioxidant and exogenously administrated has radical scavenging potential (Ohtaki et al. 2010). Therefore, it is possible that moderate harmful ROS potential (initially lower due to the shorter exposure period) was finally inactivated by PACAP-38, leading to the amelioration of the basic movement parameters. The neuromasts, as a part of the lateral line are, inter alia, responsible for a variety of behaviors including school swimming, prey detection, predator avoidance, and sexual courtship (Ghysen and Dambly-Chaudiere 2004), so their functional impairment influences the quality of the social life of fish. Moreover, it has been proven that after cellular hair cell damages induced by various ototoxins, zebrafish can experience wide range of behavior deficits (Buck et al. 2012; Suli et al. 2012; Olszewski et al. 2012) and, as found in the present study, also H_2_O_2_ at specific doses effectively and selectively affects neuromast hair cells. It has been reported that lateral line hair cell disruptions cause inability in current water flow orientation (Buck et al. 2012), significant attenuation of the innate startle, impairment of the avoidance responses, (Buck et al. 2012), and that damages to stereocilia bundle integrity disrupt rheotaxis (Suli et al. 2012; Olszewski et al. 2012). Moreover, the swimming behavior of zebrafish as a biomarker for ototoxicity-induced hair cell damage is already in use (Niihori et al. 2015). Therefore, based on the whole data obtained in the present study, it could be said that H_2_O_2_ toxicity is predominantly directed toward neuromasts and PACAP-38 performs ameliorating effect. However, it should be emphasized that impairment of the movement may not only be the result of neuromast disturbances, because it can be caused by several other factors including muscular or nervous system toxicity.

In conclusion, the present study has disclosed the protective effect of PACAP-38 on oxidative stress damage in zebrafish hair cells. Our in vivo findings reaffirm results of previous in vitro investigations suggesting PACAP-38 ability to prevent hair cells from apoptosis. We revealed that PACAP-38 treatment decreased the cleaved caspase-3 level in the hair cells, but somewhat unexpectedly had no influence on p-38 MAPK pathway. The behavioral analyses validated the PACAP-38 protective role, demonstrating that PACAP-38 treatment rescued H_2_O_2_-induced reduction in movement. In our opinion, the results obtained contribute to the knowledge, which hopefully will allow the design of potentially new-generation antioxidant drugs that could be used for the treatment of oxidative stress damaged hearing. Moreover, another, not less important, advantage of present study seems to be an obvious conclusion that the zebrafish should be considered as an excellent research tool for studying the auditory system and that the resulting findings can be treated as fully related to those obtained in mammals.
